# Supplementary material for: The Influence of Formulation Components and Environmental Humidity on Spray-Dried Phage Powders for Treatment of Respiratory Infections Caused by Acinetobacter baumannii
Source: Pharmaceutics. 2021 Jul 28;13(8):1162. doi: 10.3390/pharmaceutics13081162 (PMC8401170; doi:10.3390/pharmaceutics13081162)
Supplement: Supplementary file 1 [file pharmaceutics-13-01162-s001.zip › pharmaceutics-1259950-supplementary.pdf]

pharmaceutics

MDPI

# Supplementary Materials: The Influence of Formulation Components and Environmental Humidity on Spray-Dried Phage Powders for Treatment of Respiratory Infections Caused by *Acinetobacter baumannii*

**Wei Yan, Ruide He, Xiaojiao Tang, Bin Tian, Yannan Liu, Yigang Tong, Kenneth K.W. To and Sharon S.Y. Leung**

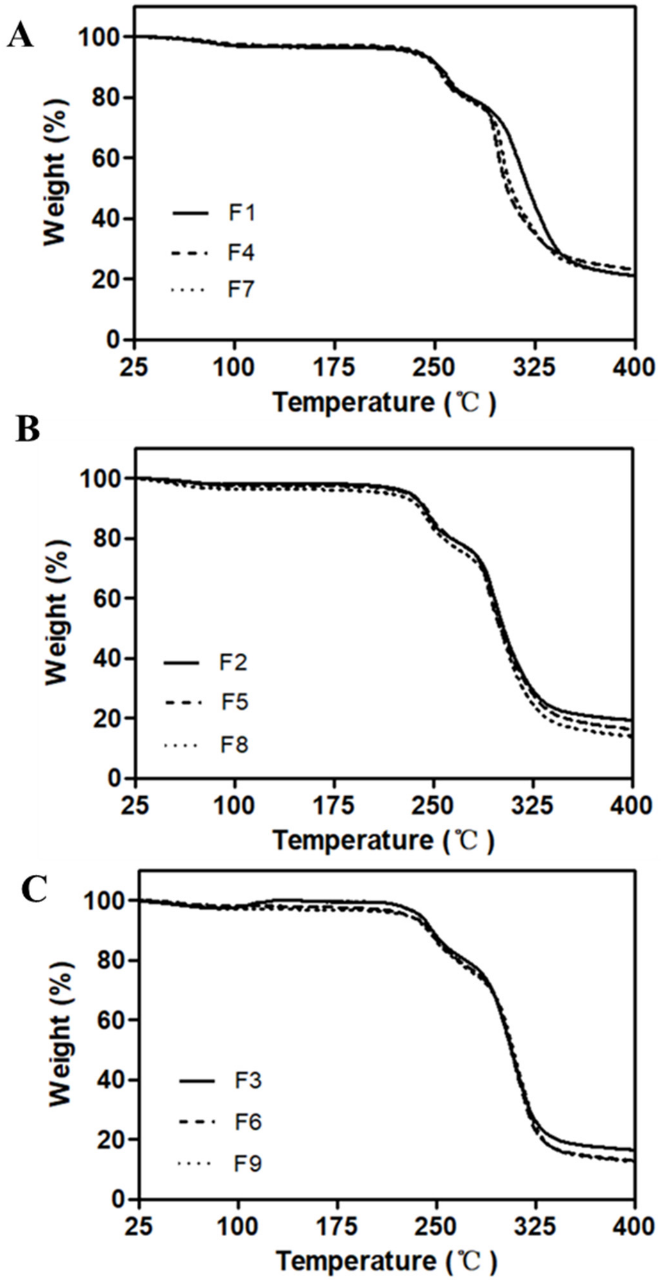


**Figure S1.** TGA profiles of the spray dried formulations. (A) F1, F4 and F7 at a formulation composition of 80% trehalose, 20% leucine; (B) F2, F5 and F8 at a formulation composition of 60% trehalose, 20% mannitol and 20% leucine; and (C) F3, F6 and F9 at a formulation composition of 40% trehalose, 40% mannitol and 20% leucine.

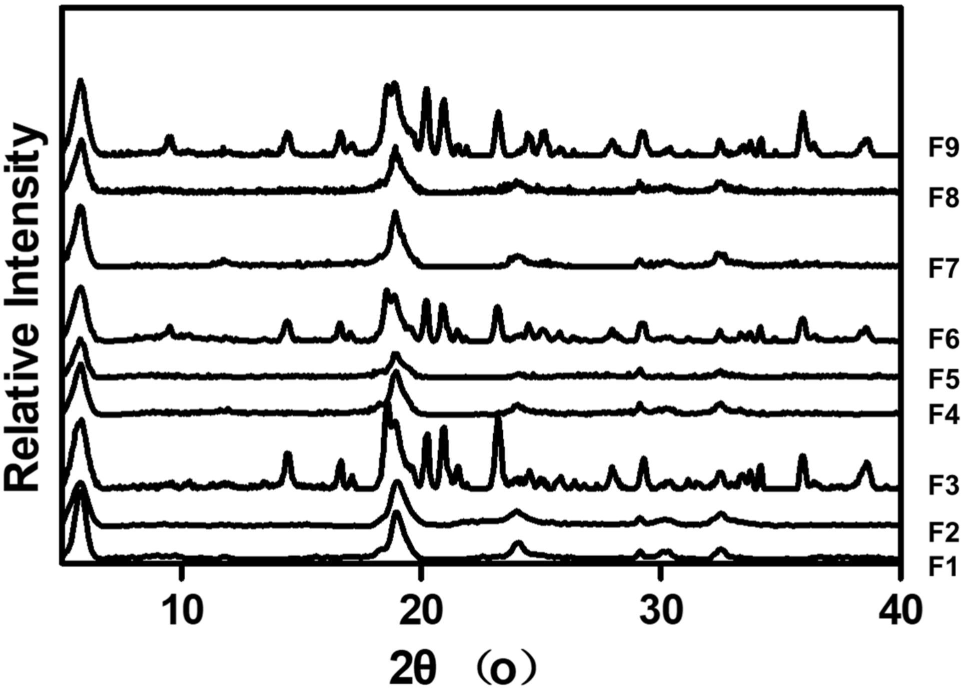


**Figure S2.** XRD profiles of the spray dried formulations.

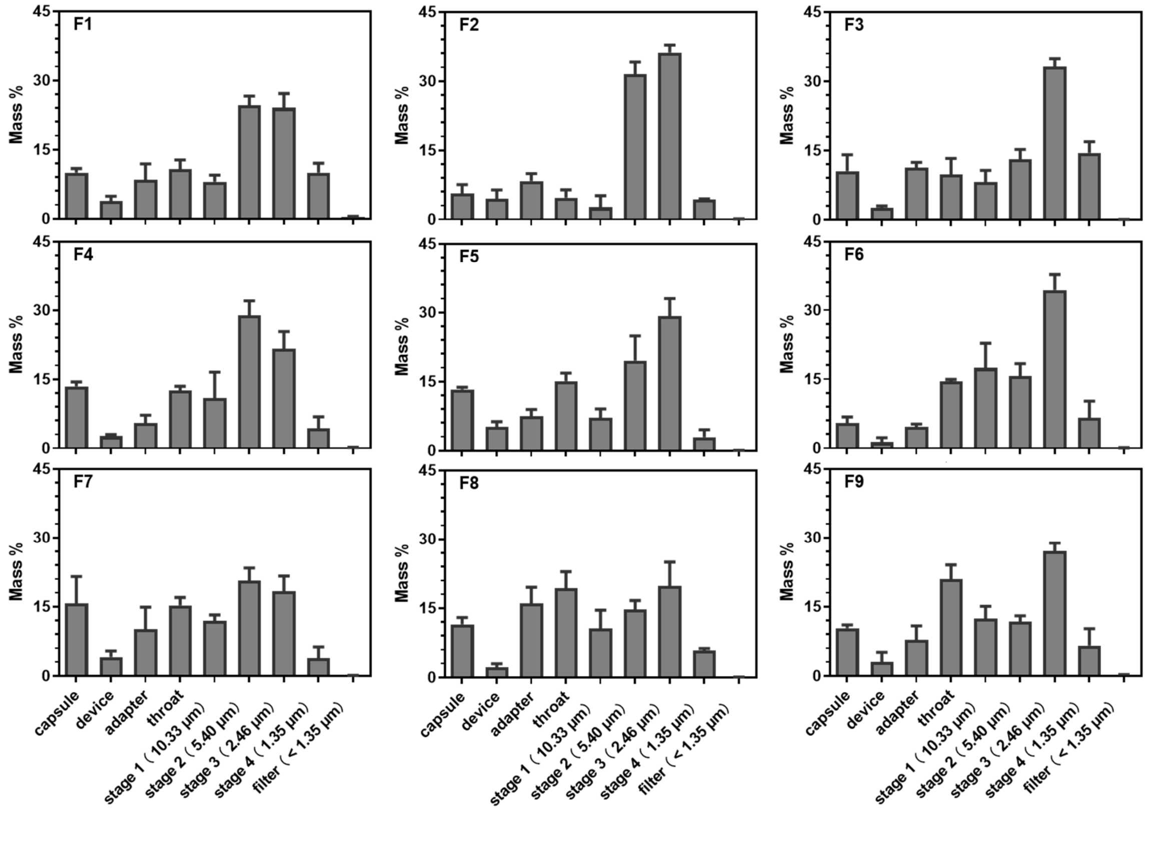


**Figure S3.** The distribution profiles of viable AB406 phage. Data presented as mean ± one standard deviation (n=3). All formulations were dispersed at 95 L/min for 2.5 s using the Osmohaler™. The aerodynamic cutoff diameter of each stage is quoted in parentheses.

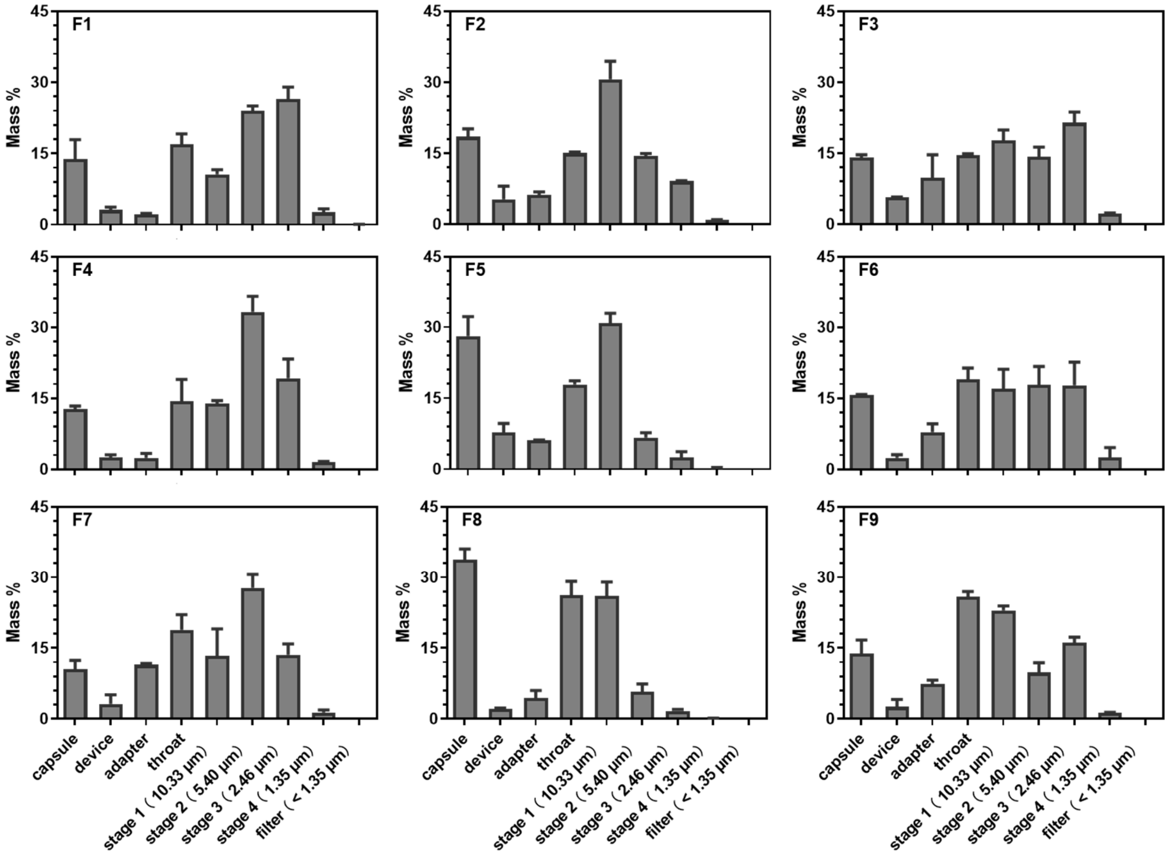


**Figure S4.** The distribution profiles of viable AB406 phage under 65% RH. Data presented as mean ± one standard deviation (n=3). All formulations were dispersed at 95 L/min for 2.5 s using the Osmohaler™. The aerodynamic cutoff diameter of each stage is quoted in parentheses.
